# Artificial Intelligence-Based Wearable Robotic Exoskeletons for Upper Limb Rehabilitation: A Review

**DOI:** 10.3390/s21062146

**Published:** 2021-03-18

**Authors:** Manuel Andrés Vélez-Guerrero, Mauro Callejas-Cuervo, Stefano Mazzoleni

**Affiliations:** 1 Software Research Group, Universidad Pedagógica y Tecnológica de Colombia, Tunja 150002, Colombia; 2 School of Computer Science, Universidad Pedagógica y Tecnológica de Colombia, Tunja 150002, Colombia; mauro.callejas@uptc.edu.co; 3 Department of Electrical and Information Engineering, Polytechnic University of Bari, 70126 Bari, Italy; stefano.mazzoleni@poliba.it

**Keywords:** robotic exoskeletons, wearable devices, artificial intelligence (AI), artificial neural networks (ANN), adaptive algorithms, upper limbs, rehabilitation, healthcare, control strategies

## Abstract

Processing and control systems based on artificial intelligence (AI) have progressively improved mobile robotic exoskeletons used in upper-limb motor rehabilitation. This systematic review presents the advances and trends of those technologies. A literature search was performed in Scopus, IEEE Xplore, Web of Science, and PubMed using the PRISMA (Preferred Reporting Items for Systematic Reviews and Meta-Analyses) methodology with three main inclusion criteria: (a) motor or neuromotor rehabilitation for upper limbs, (b) mobile robotic exoskeletons, and (c) AI. The period under investigation spanned from 2016 to 2020, resulting in 30 articles that met the criteria. The literature showed the use of artificial neural networks (40%), adaptive algorithms (20%), and other mixed AI techniques (40%). Additionally, it was found that in only 16% of the articles, developments focused on neuromotor rehabilitation. The main trend in the research is the development of wearable robotic exoskeletons (53%) and the fusion of data collected from multiple sensors that enrich the training of intelligent algorithms. There is a latent need to develop more reliable systems through clinical validation and improvement of technical characteristics, such as weight/dimensions of devices, in order to have positive impacts on the rehabilitation process and improve the interactions among patients, teams of health professionals, and technology.

## 1. Introduction

The proper functioning of the limbs of the human body plays a fundamental role in people’s health. When these limbs are temporarily or permanently affected, significant motor difficulties appear. Currently, there is a rapid growth of disability-related diseases worldwide. In a global context, the World Report on Disability [1] highlights that approximately 15% of the world’s population has some form of motor disability and 4% of them suffer from diseases linked to motor or neuromotor dysfunction. Any form of disability, whether mild, moderate, or severe, impairs an individual’s functional autonomy and interaction with the environment. Other underlying social or demographic factors may increase the incidence of disability [2], as may inadequate coverage of traditional or technology-based rehabilitation services, poor coordination of care facilities, and overburdening of existing specialists [3].

When it comes to the most common problems that trigger neuromotor impairment, stroke is recurrent in all populations [4]. Its various manifestations and consequences (e.g., hemiparesis/ hemiplegia, traumatic brain injury, and cerebral palsy) represent some of the main causes of disability in the upper limbs in the medium and long term [5,6].

From a rehabilitation perspective, the effectiveness of a traditional treatment depends on the skill of therapists, their previous experience in treating similar cases, and their ability to formulate successful rehabilitation plans [7,8]. Usually, the assessment of the patients and their progress is not quantified in a timely, adequate, and objective manner, thereby reducing the possibility of knowing the impact of rehabilitation [9].

Although the application of assessment and therapeutic systems based on robotic exoskeletons started in the past two decades [10] and has shown encouraging results in the rehabilitation of upper limbs [11,12], there are still applied research niches that deserve to be explored [13]. The design of rehabilitation technology is usually not followed by full or partial clinical trials; thus, such developments do not find direct applicability in medical or rehabilitation centres [14], which is important to pave the way for an implementation in the clinical practice.

This offers a new opportunity for the development of robust and reliable systems, enabling the recovery of lost motor control due to accidental injury or illness [15,16]. The use of active devices in rehabilitation was proved to be feasible [17], with direct benefits limited not only to patients with motor or neuromotor injuries, but also in other areas where human movement is critical and in terms of optimisation of healthcare resources [18].

The implementation of computational techniques based on artificial intelligence (AI) embedded in robotic exoskeletons for rehabilitation, and the development of lighter, portable, and ergonomic systems [19,20], represent the main topics for the present review. An active search of recent literature to underpin the future of research into this type of technology is necessary, which led to this document.

This systematic review includes articles published in Scopus, IEEE Xplore, Web of Science, and PubMed between January 2016 and November 2020. The focus of the review is on wearable and ergonomic robotic exoskeletons for the upper limbs, whose control, data collection, or processing systems are based on AI algorithms. A large number of studies report an increase in the use of different artificial neural network (ANNs) architectures, and the mixing of traditional control techniques with intelligent or adaptive optimisers, creating robust or hybrid systems.

The use of AI-based techniques has been recognised as able to enrich the rehabilitation process by providing a comprehensive assessment of a patient’s performance and increasing the confidence of final users (i.e., patients and healthcare specialists) when interacting with robots for rehabilitation.

As far as the mobility of these devices is concerned, the current trend is towards reductions in weight and dimensions to promote performance in activities of daily living (ADLs) and therefore increase independence, but it was necessary in some cases to reduce the degrees of freedom (DoF) in order to have more compact systems.

## 2. Methods

This section describes a detailed methodology used to conduct the literature review, which was driven by the guidelines described in the PRISMA method: Preferred Reporting Items for Systematic Reviews and Meta-Analyses [21]. An application of the PRISMA guidelines allows one to carry out a systematic review of the literature based on specific inclusion criteria and helps both authors and readers to make proper judgments on the reported studies.

### 2.1. Eligibility Criteria

Eligibility criteria included articles: (i) written in English, (ii) published between January 2016 and November 2020, (iii) reporting the use of robotic exoskeletons on the upper limbs, (iv) for use in rehabilitation, (v) implementing artificial intelligence-based processing or control systems, (vi) reporting mobile, portable, or wearable devices, (vii) found in the field of engineering, computer science, or medicine, and (viii) written in a paper, review, or conference paper form, with full-text availability.

With regard to the (ii) eligibility criterion, the timeframe was shortened to the last five years due to the rapid technological advancements in the development of robotic exoskeletons for upper limb rehabilitation.

It is noted that the development of applied AI-based techniques in this field started at the end of 2015, which represents the first point for looking in closer detail at the trend that will guide future research.

### 2.2. Search Methodology and Scope

For the initial information collection, a search of the reported literature was performed in Scopus, IEEE Xplore, Web of Science, and PubMed databases. Search queries were formulated with multiple keywords falling within topics already described in the eligibility criteria, and applied to the title, abstract, or keywords fields.

The keywords were: (i) Assistive technologies: “powered exoskeleton”; “robotic exoskeleton”; “active exoskeleton”; exosuit, “powered orthosis”; “robotic orthosis”; “active orthosis.” (ii) Device type: “wearable”; “mobile”; “portable.” (iii) Control technologies: “intellig*”; adaptive”; “net*”; “artificial neur*”; “ANN”; “learn*”. (iv) Body segment: “upper limb*”; “arm”; “forearm”; “shoulder”; “elbow.” (v) Medical application: “rehabilitation”, “physical therapy”; “impairment”; “health.” Table 1 presents the search queries used in each database.

### 2.3. Inclusion Criteria

The keywords used in the search stage led to the documents for the systematic review. However, the documents obtained were screened to ensure that all eligibility criteria were met. For example, documents that were not accessible in full text and documents that were duplicated in different databases were removed, as detailed below.

**Figure 1:**
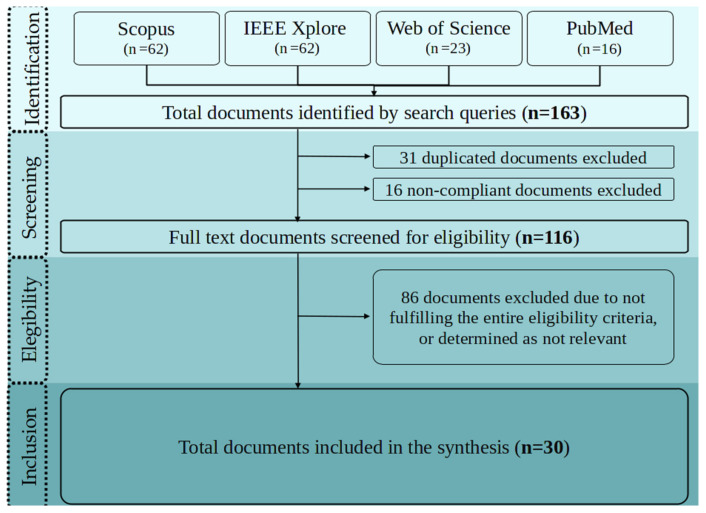
Searching, filtering, and selection of papers to be included in the review, following the PRISMA methodology [21].

The systematic review process begun with the identification of 163 documents in the search stage. The after the screening stage, 31 duplicate documents were removed, along with 16 additional documents that were not full-text nor the proper text type. Finally, 86 documents were removed, as they did not fit the objective of this review. This process resulted in the inclusion and analysis of a total of 30 documents, which are presented in this research. Figure 1 summarises the process of searching, screening, eligibility, and inclusion, while applying the PRISMA methodology [21].

## 3. Results

**Figure 2:**
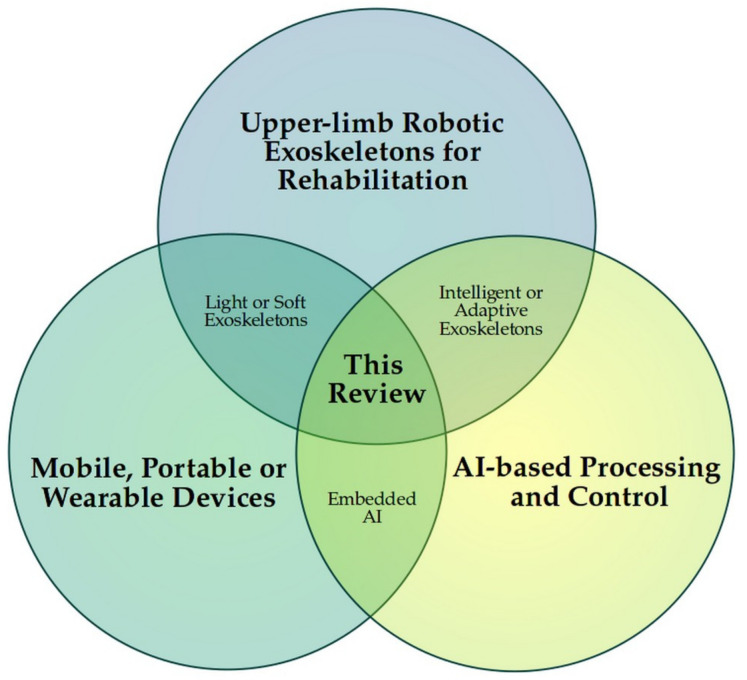
Graphic summary of the main axes that constitute this systematic review.

The results section contains the presentation and analysis of the selected documents. A narrative style is adopted to present the main conclusions, and additional information on the characteristics of the studies, the goals reached, and their contributions. In a general way, as summarised in Figure 2, the purpose of this document is to collect and show the advances and trends for the development of wearable and portable robotic exoskeletons used for the rehabilitation of patients with upper-limb motor impairments, directly involving AI-based processing and control systems.

**Figure 3:**
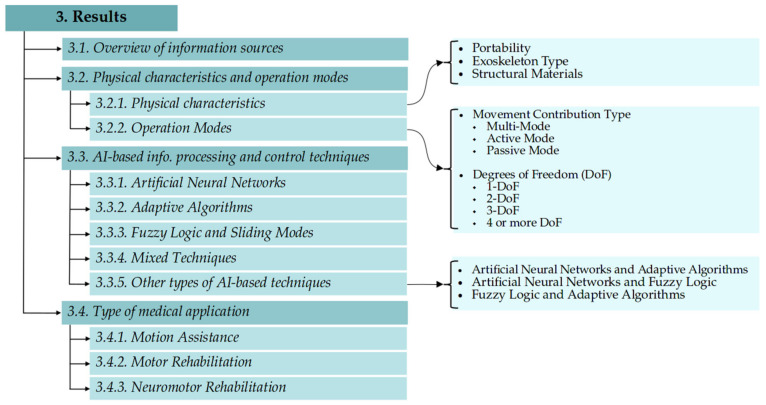
Hierarchical grouping map for the elements taken into account within the review, as set out in the following sub-sections.

This article has reviewed the final 30 articles with four different approaches: (i) a general description of the information sources; (ii) technical characteristics of the device: weight, dimensions, portability, and materials used in its manufacturing; (iii) information processing and control techniques based on AI; and (iv) the nature of the medical application on the upper limb. Figure 3 shows the structure of the presentation of the results.

### 3.1. Overview of Information Sources

Table 2 presents a summary of relevant characteristics of the 30 studies included in the review, corresponding to: bibliographic reference of the document (Ref.); publication year (Year); exoskeleton manufacture (MF); classification according to design and development type: made by the researchers (Dev) or commercial products (Com); exoskeleton type (hard: designed with rigid physical elements such as metal (ME), plastic (PL), or 3D printed plastic (3DPL); soft: without rigid structures, or semi-hard structures); portability (semi-mobile, mobile, or wearable); AI techniques used; modes of operation (active (AC), active–passive (AP), resisted (RE), and other (OT)); type of medical application (active assistance (AA), motor rehabilitation (MR), neuromotor rehabilitation (NMR)); and DoF-joint information: specific joints of the upper limb that are assisted, including the degrees of freedom for each joint.

It is important to note that Table 2 may not contain information on other characteristics of the studies reviewed, such as details about the particular design or performance of a system, or the validation level of the device in clinical trials. This is because full explicit information may not exist within all the reviewed documents, and each particular case has been described in more detail in the following sections of this document.

On a preliminary basis, most research articles reported the development of robotic exoskeletons, representing 87% of the studies, whereas 13% of final documents reported the usage of commercial developments. Besides, the recent development of soft robotic exoskeletons (6.3%), able to provide a new way of transferring motion by means of non-conventional structures, is highlighted.

**Figure 4:**
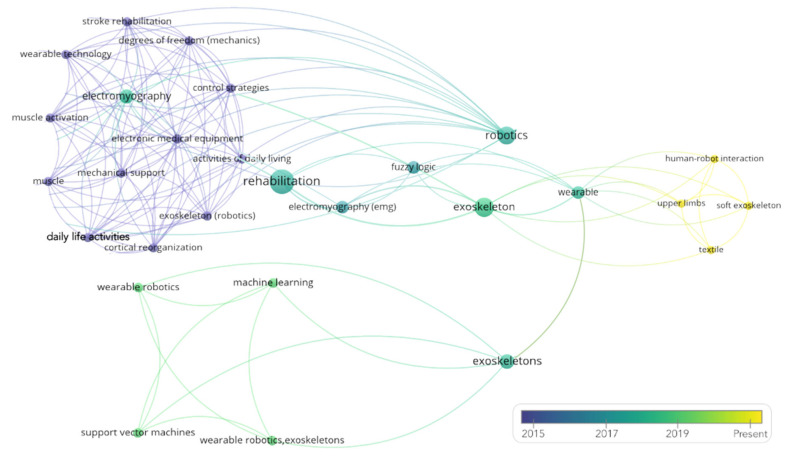
Time evolution and interconnections of the reviewed topics. Map generated with VOSViewer [56].

Finally, Figure 4 presents a general map of the key terms associated with the documents examined, revealing the evolution over time of the topics related to this research and their connections with the previously established search criteria.

### 3.2. Physical Characteristics and Operation Modes of the Robotic Exoskeletons

Robotic exoskeletons have different design features that highlight their importance for inclusion as technology for assessment and treatment. Within these characteristics, those inherent to the design and construction of the device stand out, from the choice of suitable materials to factors such as weight, portability, and the developed exoskeleton type. The characteristics related to the functioning of the device are highlighted, referring specifically to exoskeleton contributions in the movement and the range of motion in the upper limb of the human body. The characteristics described are some of the physical properties displayed in the developments, and the operating modes. These elements are detailed below.

#### 3.2.1. Physical Characteristics

**Figure 5:**
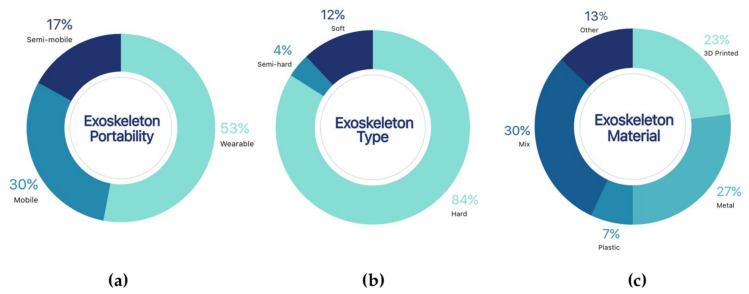
The general distribution of physical properties of the robotic exoskeletons in terms of (**a**) portability, (**b**) exoskeleton type, and (**c**) structural material if applicable.

The physical characteristics of robotic exoskeletons determine their applicability in the healthcare field. As mentioned in several studies, a compact, lightweight, and aesthetically pleasant robotic rehabilitation system is attractive for use in the clinical setting [57]. Although the mechanical design of the structure is important for the adoption of devices in the healthcare field, it would be also desirable to have increasingly robust features, regardless of the limb involved [58]. Figure 5 shows the overall distribution of physical properties of robotic exoskeletons summarised in three key assessment criteria.

##### Portability

Within the reviewed literature, the wearable exoskeletons were particularly noteworthy, representing 53% of the documents included (see Figure 5a). In this segment, among the exoskeletons with the highest degree of portability, there were some cable operated devices [50,51] wherein most of the structure is flexible and lightweight. The paper of Samper-Escudero [50], pointed out that the developed exoskeleton is anatomically adaptable based on the height of each patient. On the other hand, Varghese’s [51] development highlighted the lightness of the device, weighing approximately 950 g.

In both cases, the logic and activation support were carried out in an ergonomic backpack and not directly on the upper limb. Other developments that reported cable-based actuators [49] do not have the same mechanical qualities due to their larger structures.

In general, robotic exoskeletons that reported the use of 3D printed structures are usually characterised by their low weight and good mechanical properties that allow their use in rehabilitation. Within this segment, the developments with the highest degree of portability were reported in [22,26,29,42]. They are distinguished by the easy donning and doffing procedures and their versatility in the applications of different rehabilitation protocols. Specifically, in [22], the emphasis was placed on the weight of the wrist orthosis (330 g).

In [28,32], light, compact, and portable structures were presented. The common feature of these developments was the simplicity of the structures (resembling 3D printed structures) without neglecting ergonomics and functionality, key aspects to performing effective motor function rehabilitation.

In detail, some documents [33,37] showed an excellent blend of high-performance features and portability. These exoskeletons ensure maximum portability while maintaining a variety of ranges of motion with a robust kinematic design. Due to their lightweight structures, it is possible to have one of them on the user’s shoulder or back. Specifically, in [37], it was pointed out that the structure is compact and light, which allows the user to wear it by means of belts.

Guo et al. [40] points out that “the portability, lightweight and easy operation (of the proposed exoskeleton) make the patient only need to wear it on the limb to assist in the performing of rehabilitation training.” However, the device can be improved through static analysis, overcoming the disadvantages of the structural weight.

The same robotic structure was used in three different documents [39,43,47]. The system reported by these investigations presents an adequate level of portability and a comparatively light weight (1.6 kg) for use mainly at the elbow joint. This type of robotic exoskeleton features kinematic modelling that supports the rehabilitation process. Similar developments [23,25] do not directly show the proposed exoskeleton for rehabilitation or research, but as deduced from the narrative, they consist of portable and wearable systems with sufficient degrees of mobility for motion assistance processes in medical applications.

Complex and wearable or mobile structures are shown in [24,31,41,55]. These devices were noteworthy for maintaining compact properties of usability and strength, despite being bulkier structures. The work carried out in [24] has highlighted the effort put into designing a system that can be used in daily life without affecting the freedom and mobility of the patient. However, the total weight is around 4 kg.

Other robotic exoskeletons of a mobile nature account for 30% of the reviewed studies. Although they are portable and are not in need for fixed structures in the operation, they require a longer setup time due to their complexity, dimensions, or weight. Therefore, mobile exoskeletons are candidates for use in physical recovery only within a controlled rehabilitation environment. An example is reported in [27], where although the exoskeleton shown has excellent technical characteristics, its weight being close to 20 kg limits its usability outside the appropriate testing and training environments. In the same vein, some other developments with mobile exoskeletons were presented [36,38] providing mid-level characteristics in terms of complexity and mobility. Within these studies, it should be noted that the kinematic design of each prototype is complex, and therefore a mechanical optimisation is still required.

Finally, some studies reporting semi-mobile devices were found (17%). These devices require additional support structures, limiting patient mobility in different environments, and therefore the versatility of the proposed device [34,48,52,53,54,59]. These exoskeletons were some of the pioneering devices wherein several improvements were carried out in order to improve portability and the consequent autonomy of each patient.

The advances shown in [46] have provided a look into the future: intelligent exoskeletons with lightweight structures should be preferred to allow maximum patient mobility, considering also improved designs towards increased ergonomics and appropriate materials to achieve lower weight. This step is rather important to accelerate the adoption of robotic exoskeletons in the rehabilitation domain.

##### Exoskeleton Type

The most frequently developed type of robotic exoskeleton is the hard exoskeleton, corresponding to 84% of the reviewed literature (see Figure 5b). Rigid exoskeletons are systems designed mechanically to support the full weight of the limbs [31,36,40,45,52,53] and their structure usually offers high resistance to tension or deformation [23,24,41,48]. Hard exoskeletons can be quite compact and provide some mechanical flexibility, which facilitates limb mobilisation tasks [22,29,32,33,37,42,46].

Some of the developments shown in this literature review corresponded to rigid bilateral systems [27,34,49,55], designed specifically for synchronous motor rehabilitation. Some of them proposed different methodologies to detect the movement intentions and to replicate them using a leader–follower architecture. Although hard exoskeletons have been traditionally accepted [39,43,47,54], several alternatives have proposed improvements in terms of weight or functionalities [30,35,44].

Other developments were reflected in the 12% of the reviewed literature corresponding to soft exoskeletons, which lack a rigid structure. Soft–type exoskeletons [50,51] promote a new generation of attrition-resistant robots that use anatomical body structures. A flexible and soft device was highlighted in [50] to assist the user’s natural movement without any restrictions.

Finally, there are some minor advances (4%) that report the use of soft structures added to some features of completely rigid exoskeletons. For example, an effective passive orthosis design was shown in [28], featuring properly tensioned viscoelastic bands for a tight fit, wherein the torque was directed to the wrists to provide a maximal mechanical effect.

##### Structural Materials

As regards the structural materials used in the reviewed devices, 30% of the developments showed mixtures of two or more materials in their structures (see Figure 5c). The addition of plastic elements to the metal structures that improve the physical appearance and reduce the weight represents the most common form of combination [23,34,46]. In developments such as [40], ANSYS static analysis was also used to examine the maximum strength and pressure of the mechanical structure, thereby helping to select the best materials. Other developments focused on the standout designs [37], such as the perfectly integrated combinations of different materials. In addition, the design of the structure, regardless of the construction materials, was generally adaptable to match the different anthropometric sizes of the patients.

It is also common to find structures formed by a wide variety of metals (27%), which provided rigidity to the structures [27,36,41,45,52]. For instance, in [31] after numerous tests it was concluded that the most appropriate material to be used is aluminium alloy 6061-T6. Despite the all-metal structures, in [24,49] the proposal of new cable-operated joints was highlighted, contributing to making the exoskeleton light, compact, and portable. Finally, as described in the portability section, the same structure reported in three papers [39,43,47], was composed of metals and a minimum number of other elements. In order to reduce the overall weight and ensure the required strength, the main structure was composed of 5052 aluminium alloy. It should be noted that the appropriate materials to design the exoskeleton were carefully selected, ensuring the durability.

With the rise of additive manufacturing and 3D printers, a growing number of developments have reported the use of fully printed structures, using for example acrylonitrile butadiene styrene (ABS), polylactic acid (PLA), or nylon type plastic parts, representing 23% of the literature. As a specific case, in [22,29,33], the main feature is the simplicity of the mechanical design without affecting the intended rehabilitation function. These characteristics have allowed caregivers to implement different rehabilitation protocols for elderly populations or patients with motor impairments. Other devices are less compact but provide high levels of support [42,48,53,54,55]. For the system by Bakri [42], simulations and resistance tests were carried out to confirm the proper functioning of the material with multiple weights on the mechanical structure. Taulman nylon 680 and polyethylene terephthalate glycol-modified (PETG) plastic were used, which have good mechanical properties and low cost, and meet the FDA’s regulatory standards for use in medical devices.

Finally, a small percentage of studies reported the use of different types of soft or semi-rigid materials [32,50,51], corresponding to 13% of the reviewed literature; thermoformed plastic currently represents only a small portion (7%). In particular, in the orthosis shown in [28], materials such as thermoplastic urethane (TPU) were used, providing durability and flexibility to the design.

#### 3.2.2. Operation Modes

**Figure 6:**
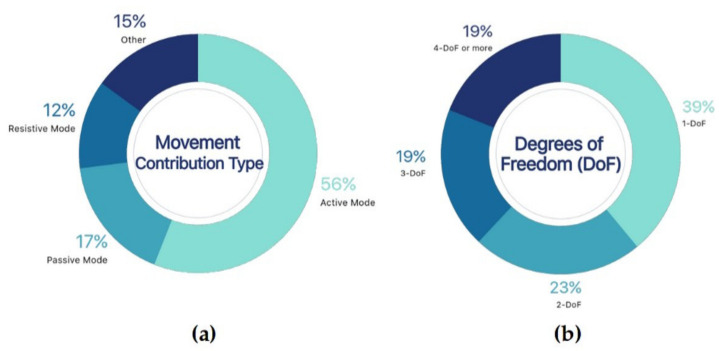
The distribution of the operating modes of robotic exoskeletons in terms of (**a**) movement contribution types and (**b**) total degrees-of-freedom (DoF).

The characteristics related to the functioning of robotic exoskeletons reported in the literature can be clustered according to the movement contribution type and the number of degrees of freedom (DoF). Figure 6 shows the distribution of the operating modes.

##### Movement Contribution Type

In the rehabilitation of motor or neuromotor function, four control modes are generally considered: (i) active mode, (ii) passive mode, (iii) assisted mode, and (iv) resisted mode [60]. The active control mode provides all necessary movement to the limb by the robotic exoskeleton, which represents 56% of the studies reviewed (Figure 6a). In our sample, 17% of the literature has reported the use of passive control mode, which refers to the accompaniment of the movement produced by the limb using an exoskeleton. The assistive mode (15%) helps the movement of the limb partially, and the resisted mode offers opposition to the movement performed by the limb, representing 12% of the literature.

Each of these monitoring modes is brought into rehabilitation systems and provides a range of medically quantified outcomes, as each monitoring mode emphasises a specific type of recovery [61]. Control modes can be complementary to each other for rehabilitation exercise using robotic exoskeletons [62]. It is worthwhile to mention that in the examined developments, two or more operation modes can coexist in the same device: this topic will be discussed in detail below.

- Multi-Mode

Documents such as [30,35,44,46,55] showed progress for several devices using mixed modes of operation, distributed almost proportionally. Some advantages of each mode were highlighted: (i) In passive or resisted modes, if the patient is active during the therapy session, a better environment for rehabilitation treatment is offered. Therefore, the exoskeleton with the patient-driven control strategy produced significantly better results. (ii) Partially assisted modes of operation implemented through interactive strategies encourage user participation and promote greater effectiveness of rehabilitation. (iii) The active operation mode incorporates task-oriented functions. These exoskeletons are not limited to rehabilitation processes, but can be used to increase human performance.

The robotic orthosis presented by Sangha [22] was designed to function in four modes: passive, active, resisted (helping users who lack muscle tone or do not have full motion range), and active-associative (helping users to increase interaction and decrease muscle abnormalities). In this work, an experimental protocol was developed in which a healthy volunteer tested the three operation modes. The results showed that the robotic orthosis was able to successfully operate under any functional parameter.

In [23], six modes of operation were presented: passive, passive stretching, passive guidance, active initiation, assisted active, and resisted active. It was noted that the proposed system can be used by various kinds of patients, for example, a patient without any muscle strength or a healthy person who has temporary muscle deficiency problems.

Finally, the development shown in [49] has provided different modes of operation, such as patient-active-robot-passive and patient-passive-robot-active training, achieved based on trajectory tracking control.

- Active Mode

Various developments [24,25,26,31,42] do not specifically refer to the implemented operation modes; however, it was deduced that an active control was implemented. In other works [39,40,43,45,47,51], the robotic exoskeleton has provided complete rehabilitation assistance through active control.

Complex systems have implemented an active operation mode [27,32,41,48,50], which was focused on providing full assistance to the affected limb. The support to the upper limbs is triggered by the measurement of the activity in the electroencephalography (EEG) or electromyography (EMG) systems, and in some cases by eye-tracking data.

As regards compact active exoskeletons, in the paper of Lambelet et al. [29] an electrically actuated exoskeleton for rehabilitation of the wrist joint was presented. For its part, the development shown in [34] has highlighted the use of the active operation mode where Series Elastic Actuators (SEA) able to reduce the inertia and intrinsic impedance of the actuator, provide a more precise and stable force control.

Finally, [28] provided a semi-active type of assistance; the physical design was intended to mitigate involuntary pronation-supination tremors in the upper limb. This paves the way for passive control mode robotic exoskeletons.

- Passive Mode

Exoskeletons with a passive operation mode are appropriate to support upper limbs of the patients as they eliminate the gravitational load and provide the user with the opportunity to perform the necessary rehabilitation tasks. Under this umbrella, the studies [33,38] were presented, wherein it was emphasised that these kinds of devices are able to relieve the load on the limb to be treated when placed in a real environment. Similarly, in [36] different controllers were designed to perform the passive operation mode within the developed exoskeleton.

In more comprehensive works, the different capabilities of the passive mode are explored [37], focusing on the intentions of the user’s natural movements and avoiding a direct interaction with the rehabilitation system. In this research, the exoskeleton could be applied in the rehabilitation training and provide support to the ADLs.

##### Degrees of Freedom (DoFs)

The DoFs of the robotic exoskeleton determine the possible movements over one or multiple joints of the upper limb. As a general rule, the higher the number of degrees of freedom, the more actuators are needed [63]. This leads to an increase in the complexity of the mechanical design, and the control systems to properly execute the different movements [64]. In different studies, more than three passive DoF were implemented, so a detailed examination is required.

- One DoF

The articles that present one DoF are rather abundant, representing 39% of the reviewed literature (Figure 6b). These studies have focused on any movement of the different joints of the upper limb. The devices that operate on the wrist joint were included [22,28,29,32]. In some of these articles, an increase in the number of DoFs of the robotic orthosis was proposed as future work. This would make it possible to include, for example, other wrist movements (i.e., adduction/abduction and prono/supination) and finger movements.

The use of a single DoF simplifies the design process in terms of driving mechanisms that allow proper joint mobility, but increases the complexity of the controller.

Other works characterised by one DoF were presented in [41,45,46]: they emphasise the movement of the elbow joint. In general, these developments are able to assist the movement of the joint for both flexion and extension movements. In [48] it was shown that the two fixed forearm blocks slid on a linear rail to provide the forearm DoF, allowing the exoskeleton’s joints to synchronise with the patient’s joints.

- Two DoFs

The articles featuring a design with two DoFs constituted 23% of the reviewed literature (Figure 6b). Indeed, two-DoF modelling is quite common [25,50]; shoulder and elbow joint movement prevail. In [34] the constant alignment between the user’s elbow and the axes of the exoskeleton during movement was performed by means of passive mechanisms that can avoid the translational forces caused by the misalignment of the joints.

On the other hand, [36] presents the structure of an upper-limb exoskeletal robot with two DoFs in the upper limbs, where the physiological characteristics in different phases of a clinical condition were analysed to determine the condition of the patient. It was concluded that the practical effect of rehabilitation training can be improved by changing the various mechanical structures.

- Three DoFs

The developments that presented designs with three DoFs constituted 19% of the reviewed literature (Figure 6b). Specifically, the exoskeleton used in [39,43,47] established a DoF at the elbow joint, a DoF at the forearm rotation, and a DoF at the wrist joint. The main objective of this exoskeleton was to provide rehabilitation treatments with a small, lightweight, and high-performance device. Furthermore, the safety of the device, its ergonomics, and rehabilitation theory were taken into account in the design as well.

Other developments are presented in [23,42]; most articles have included a combination of several movements. Specifically, in [33], the exoskeleton showed the ability to provide elbow flexion–extension, shoulder rotation in the frontal plane, and shoulder elevation. Each DoF can be independently locked by electromagnetic brakes. In [40], the device was used to mobilise elbow and wrist joints. The mechanical framework design was composed of a rotating joint at the wrist and a telescopic joint at the elbow.

- Four or more DoFs

Articles with four DoFs or more also constituted 19% (Figure 6b). Some of these developments are of high complexity, as shown in the CAPIO robot [27]. Although the initially referenced article does not show the extended information, a previous document collected all the mechanical properties [65]. This system has (i) four active DoFs that perform the backward movement with two adjustments; (ii) three drives on the shoulder joint; (iii) a rotary elbow joint in combination with a linear compensation joint; and finally, (iv) a passive joint that connects the exoskeleton to the user’s arm.

It is remarkable how this development has paved the way for subsequent research, such as the Recupera rehabilitation systems [52,53] and the Axo-Suit [54], which offer a wide range of mobility, not only focused on the upper limbs, but also on the movement of the entire human body. The importance of multi-DoF upper limb rehabilitation robotic systems is not only in their functionality, but in the fact that in most cases, they are precursors to robotic exoskeletons with greater capabilities, and serve as a basis for the development of more robust and efficient control systems.

Although less complex but still robust, the developments shown in [24,31,37] involved five DoFs, whose main movement was centred on the shoulder joint. It was highlighted that due to the system’s complexity, the dynamic model has more restrictions when controlling its movement. In general, with five-DoF models, the range of movement of the carrier is guaranteed, and kinematic compatibility with different users is ensured. In [37], an extra DoF was included in the elbow joint.

In detail, [31] stated that an important objective was the matching between the exoskeleton and the human movements. The five DoFs were arranged as follows: (i) three DoFs for the glenohumeral joint, and (ii) two DoFs for the scapulothoracic joint. As passive joints, they added (i) the scapular retroposition/preposition movement and (ii) the elbow joint for user comfort.

An article has presented the implementation of four DoFs [49]. This system is driven by pneumatic elements, and is characterised by a safe, compact, and light structure that complies with the movement of an upper limb as closely as possible to reality. “Research efforts were also concentrated on the dynamic modelling and controller design for the reliable implementation of rehabilitation exercises. The dynamic model was developed in terms of quasi coordinates combined with the virtual work principle” [49].

Finally, the most sophisticated work was carried out by Varghese et al. [51], where the multi-DoFs shoulder kinematic detection framework was introduced. This work was developed and analysed based on the physiology and morphology of one patient and will be extended in the future for multiple patients. “Based on this work, we believe that the tendon-routing architecture can be extended to other multi-DoFs joints (such as the hip, wrist, and ankle), and also to the actuation framework of the exosuit” [51].

### 3.3. AI-Based Information Processing and Control Techniques

**Figure 7:**
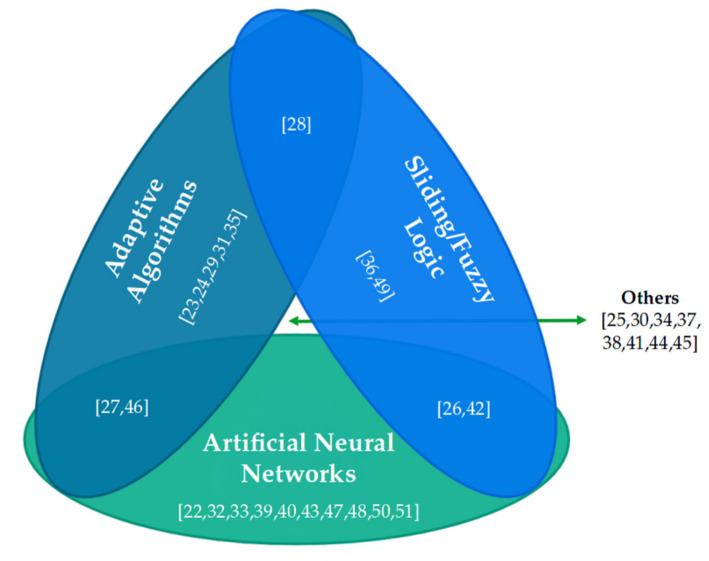
Subdivision of reviewed documents according to the type of AI-driven technique used for information processing or control strategy.

The recent use of advanced data acquisition, processing, and control techniques based on AI made possible the development of robust control strategies that can outperform classic approaches implemented in biomechatronic systems, including robotic exoskeletons [66,67]. Some of the most popular techniques are based on different configurations of ANNs, adaptive algorithms, fuzzy logic, or other techniques able to perform pattern detection or motion intention analysis. In Figure 7, the distribution of literature references that use one or more AI-based information processing and control techniques is presented.

It is established that the basis for the development of AI-based processing and control systems should aim at (i) performing an intention detection; (ii) offering modulation of the control scheme of a human–robot task (arbitration); and (iii) providing feedback during training exercises [68].

#### 3.3.1. Artificial Neural Networks

Artificial neural networks (ANNs) have historically represented one the pillars of biomechatronics and intelligent systems [69]. Through ANNs, the deployment of intelligent medical assistance devices has increased, such as prostheses operated by brain-machine interfaces and more sophisticated robotic exoskeletons that support rehabilitation tasks [70].

Within the studies included in this systematic review, ANNs take a central role: in fact, 33% of the articles show the exclusive use of ANNs, which represent the main intelligent technique for the processing and control of wearable robotic exoskeletons for the rehabilitation of the upper limbs.

Two works from the Northwestern Polytechnical University in China [39,47] stand out in the literature review for their similarities in intelligent control strategies. Kalman filters and autonomous learning machines based on different neuronal architectures were used to detect the intentions or sudden changes in movement during the training of the patient’s upper limbs. It was concluded that the control method is satisfactory in terms of movement planning and path learning for periodic rehabilitation.

Using a similar approach, the articles [22,43,48] presented intelligent strategies with the addition of electromyographic (EMG) and force-myographic (FMG) signal processing, among others. The use of a simple ANN with backpropagation algorithm learning (BP) was proposed in these cases, which led to the development of discrete and continuous motor control models for exoskeleton motion. Labelled data were used for training, while opting for a supervised learning model. Once the BP-ANN was trained, predictions were made with unlabelled test data. Despite successful results, the implementation of fuzzy systems to reduce the training time of BP-ANN was highlighted as a further improvement [48].

Although other studies used EMG signals [32], the approaches undertaken in the development of intelligent controllers were different. The hierarchical control strategy takes into account EMG and wrist strength information. This control was implemented using a BP-ANN that was trained from exercises performed previously with other patients. As a main result, it was concluded that the control system was capable of anticipating a human action by approximately 0.2 to 0.3 s. This allowed increasing the smoothness of the exoskeleton control by creating customised maps for each patient.

Advanced implementations based on ANNs are shown in [51] where the mapping of sensor information directly to joint angles was highlighted. Considerable performance was achieved, measured using RMSEs of 5.43 and 3.65 degrees in the estimation of azimuth and elevation angles of the joint, respectively. As future work, the development of “one-shot” learning and transfer learning techniques through hysterical long-short term memory (LSTM) and ANN to generalise the learned model and reduce training times was proposed.

A radial basis function neural network was implemented as a control method in [40], which achieved mechanical deficiency compensation compared to a conventional control system, such as PID. According to the error graph, there was a maximum error range of 15°, and the accuracy was improved by 3°. The control curve versus the theoretical curve showed a good effect on the controller.

The design of the control strategy shown in [33] was also based on its outcome compared to a classical PID control strategy. To maintain the direct interaction of the robotic system with a functional electrical stimulation (FES) system, a model of an electrically stimulated human arm and an ANN-based algorithm for the test patient were trained by simulation. It was concluded that the control based on a reinforcement learning strategy overcomes the PID; numerous advantages appeared. The architecture could be used in more challenging application environments.

To close this sub-section, Samper-Escudero et al. [50], extending information about the control process presented in a previous document [71], established the feasibility of performing estimations of the shoulder joint’s position using ANNs starting from the information collected by flexo-resistive sensors: 14 different models were trained using MATLAB Machine Learning Toolbox. As a result, it was concluded that the trained pattern recognition model can be used to accurately operate the robotic exoskeleton shown in [50], reaching an adequate level of performance and adaptability for different users.

#### 3.3.2. Adaptive Algorithms

The adaptive-based algorithm techniques encompass a wide variety of intelligent control and processing system developments implemented in mechatronic applications [72]. These techniques provide fundamental support for the development of predictive and self-evolutionary control systems [73]. In the biomedical field, adaptive algorithms provide better human-machine interaction, actively modifying control parameters according to the user’s continuous performance [74]. Through them, it is possible to develop assistance strategies for the rehabilitation of the upper limbs according to the patient’s functional ability [75].

In general, adaptive algorithms represent 17% of the reviewed literature, being one of the booming methods for intelligent processing and control in rehabilitation robotic exoskeletons. Since it is a family of stochastic algorithms and not a particular technique [76], it is necessary to determine what are the characteristics of each development outlined in the literature.

The development shown in [23] made use of algorithms with adaptive characteristics, where the implementation of an intelligent control algorithm in a personal computer was described. A system was programmed based on finite-state machines that can adapt their behaviour in the presence or absence of status indicators, guiding the interoperability process between the different devices used. The ability to drive the device in various operating modes was highlighted, making the solution suitable for use in multiple patient types.

In [24] a torque compensation system was used to improve the performance of the exoskeleton. In addition to this technique, the application of gravitational balancing is implemented, which reduces the requirements of larger actuators. With other reference systems, as in the case of the Harmony rehabilitation robot [55], the use of torque compensation techniques is widespread among high-performance rehabilitation systems. Experimental results showed that this type of control has a low impedance at the joint level, imposing little resistance to the free motion of patients.

Similarly, in [29] an adaptive position control was designed based on an admittance controller that receives an input force that moves in response. It was concluded that the integration of surface electromyographic signals (sEMG) into the adaptive admittance control makes it possible to automatically adjust the assistance in relation to the input signal.

Other developments presented in the literature [31,35] has shown adaptive control systems implemented through various techniques. Researchers from the Instituto Politécnico Nacional de México [31] used a PD control method along with an adaptive gravity compensation controller, similarly to [24]. The results showed that the adaptive controller produced a smooth movement path that slowly achieves the reference, suitable for rehabilitation exercises. Lastly, Miao, et al. [35] underscored the properties of adaptive algorithms compared to other controllers in terms of temporal response and variability tolerance, which is desirable for robotic rehabilitation systems. The need for adaptive control was underlined when incorporating the temporally variable capabilities of patients.

#### 3.3.3. Sliding Modes and Fuzzy Logic

Processing and control algorithms based on sliding modes and fuzzy logic represent a small proportion (7%) of the reviewed literature. The evolution of these control techniques leads to robust systems that are being explored in mechanical devices. These algorithms find application when there is the influence of parametric uncertainties due to modelling errors, non-linearity, and external disturbances [77], conditions that may occur in robotic exoskeletons for rehabilitation [50,78].

In the literature review, sliding-mode control strategies are presented in Zhao et al. [36], where the design of an anti-interference controller to increase the monitoring accuracy of stroke patients performing passive upper-extremity training was demonstrated. It was concluded that the proposed controller was effective, (i) as it meets the requirement for accuracy in positioning the rehabilitation system, and (ii) demonstrates a high anti-interference capability. The development of more complex models based on robust controllers was proposed as a future goal.

On the other hand, fuzzy logic techniques have made it possible to improve existing sliding techniques [79], providing a higher degree of precision in the estimation of the dynamic properties of the exoskeletal system [80]. In this way, control techniques based on fuzzy logic make it possible to translate conventional logical statements (hard logic) into non-linear mapping: such techniques are able to improve the performances of various types of mechanical systems [81].

Within the reviewed documents, a system based on fuzzy rules merged sliding mode control components (AF-SMC) [49], which was designed to control the movement path in the rehabilitation process. Several handling-type tests were performed on healthy subjects to validate the behaviour of the controller over passive rehabilitation exercises. The results showed that the overall performance was adequate for the preliminary application of robotic training. In the future, model-free control methods can be considered, using for example an active disturbance rejection controller (ADRC).

#### 3.3.4. Mixed Techniques

The combination of two or more AI-based techniques is common in the reviewed literature, representing 23% of the articles. The mixed techniques enable the improvement of the characteristics of the controllers based on individual algorithms, where performance is usually measured in terms of optimisation of the parameters within the control algorithms. The mixed techniques found in the literature reviewed are mentioned below.

##### Artificial Neural Networks and Adaptive Algorithms

Adaptive algorithms provide dynamic optimisation of the parameters of a common artificial neural network to achieve greater efficiency, resulting in artificial adaptive neural networks [82].

Since ANNs traditionally learn internal representations by adjusting the weights of the network connections, the inclusion of an adaptive algorithm improves learning by retropropagation through non-linear optimisation theory. This approach is useful when the learning process of an ANN is affected by accuracy flaws in the numerical computation, and environmental changes that cause unpredictable deviations of the parameter values from the original configuration [83].

In the development proposed by Seeland et al. [27] an alternative solution to performance reduction issues in classification systems when using historical data for intelligent model training was investigated. It was possible to demonstrate that the use of adaptive ANNs contributes to achieve less variability in performance and more robustness. It was concluded that: (i) it is possible to use a mixed approach (adaptive and generalised) depending on the amount of training data available, and (ii) the result is promising for achieving sensorimotor rehabilitation of the upper body using robotic exoskeletons.

Finally, [46] shows an architecture that can be used as a basis for new developments of intelligent robotic exoskeletons. It was pointed out the use of artificial neural networks and adaptive algorithms can have a positive impact on the rehabilitation domain as (i) the use of the mixture of algorithms enables the implementation of personalised rehabilitation programs, and (ii) it turns the rehabilitation assistance robot into an expert system that can support the job of physiotherapists. This architecture resulted in intelligent exoskeletons “oriented to new generation devices for assistance in upper limb rehabilitation” [46].

##### Artificial Neural Networks and Fuzzy Logic

The interaction between ANNs and fuzzy logic-based controllers is similar to the one just reported for adaptive algorithms. Neuro-fuzzy systems are able to compute optimal values of parameters used in traditional neural networks by implementing soft rules that belong to the fuzzy domain [84].

Although the combination of these two types of intelligent processing techniques is not completely new [85,86], it provides interesting results when applied in the robotics domain [87]. The main contributions vary according to two characteristics: (i) the modelling of several structural aspects of ANN in similarity to the human brain, both in structure, reasoning, learning, and perception; and (ii) the modelling of artificial systems and related data, and their application in clustering and pattern recognition tasks, function approximation, parameter estimation of robotic systems, among others.

In the work carried out at the Universiti Teknologi Petronas [26] a non-invasive human-machine interface based on sEMG and an algorithm for estimating torques in the joints by implementing a neuro-fuzzy classification system was developed. It was noted in the study that: (i) fuzzy logic inference systems have demonstrated excellence in the process of EMG/Torque estimation in real-time; and (ii) optimisation of membership functions by expert systems have reduced training time and provided better outcomes.

Finally, in [42] EEG signals were used, being segmented and classified according to the patterns exhibited by the fuzzy ANNs to perform a precision motion in the robotic exoskeleton. Specifically, the control strategy based on this approach is able to detect the deviation of each sample by continuously tracking the inputs and merging them with an artificial vision to detect and recognise the objects on a table. As a result, it was highlighted that “the proposed system shows the potential of developing a product that would enable paralysis patients to eat, drink and perform daily activities independently, with minimum training required” [42].

##### Fuzzy Logic and Adaptive Algorithms

The combination of adaptive control and fuzzy logic systems enable updating the parameters of the fuzzy controller during the fitting process. Additionally, adaptive fuzzy control can provide an efficient method for modelling more complex non-linear systems such as robotic exoskeletons with multiple degrees of freedom [88].

The main contributions of this adaptive-fuzzy systems consist in ensuring the uniformity of the constraints of the control system, regardless of the uncertainties and disturbances affecting the exoskeleton [89]. This provides stability to all the elements that compose the model of the system. An example is clear in muscular models of non-linear behaviour with the presence of perturbations, such as spasticity or fatigue resulting from the use of robotic exoskeletons [90].

Although this approach was addressed in [49], research at the Birla Institute of Technology and Science [28] directly applied different control strategies to provide a semi-active response using adaptive fuzzy proportional-integral-derivative controllers. The authors of the study have observed that the fuzzy gain setting for the adaptive PID controller is better in almost all relevant aspects of the study, thereby proving that (i) an Absolute Integral Error (AIE) less than half of the error value of the conventional reference controller and (ii) a faster settling time in almost 1 s, taking into account a 5% tolerance, were achieved.

#### 3.3.5. Other AI-Based Techniques

Finally, other varied techniques involving artificial intelligence have a significant rate of 20% of the literature, where optimised techniques of classical controllers are predominant. As a starting point, the review by Zaroug [44], stated that the fusion of machine learning algorithms with exoskeleton controllers improve the human-machine interface and the user experience by synchronising the intention of the movement with the activation of the robotic exoskeleton. Similarly, an in-depth analysis of various intelligent control techniques for operating active rehabilitation devices was highlighted in [30], showing the trend for future research.

Specifically, in the work carried out by the University of Malaysia in Pahang [25], several types of intelligent and classical controllers were used for specific exoskeleton functions. In the proposed architecture, a PD controller was added to an intelligent force system optimised by means of a swarm of particles (AFC-PSO) to examine its effectiveness for disturbance compensation. It was established that the proposed system was better correcting the disturbances that was introduced into the system while maintaining outstanding performance compared to the classic PD controller. Similarly, a hybrid active force controller (AFC) was introduced in [45], which demonstrated the ability of the robotic system to track the path over the elbow joint with greater precision. Its operation was based on a heuristically adjusted PD controller using the above-mentioned system.

Other intelligent processing techniques are shown in [38], where an approach based on the Cross-Recurrence Plot (CRP) and Recurrence Quantification Analysis (RQA) was proposed to analyse the progression of muscular fatigue in the upper limb. Although a quantification method was established rather than a complete controller, this development paves the way for the development of robotic exoskeletons that ensure complete musculoskeletal recovery.

Finally, papers such as [34,37,41] showed other simple control methods based on AI techniques, integrating biological signals, which are able to provide the exoskeletons with good handling capabilities while extracting the main characteristics and patterns of the input signals.

Regardless of the approach and techniques selected for intelligent systems, AI-based algorithms improve data collection by providing constant and accurate feedback on the performed movement, increasing the versatility and robustness of automatic control of robotic exoskeletons for rehabilitation [91,92].

### 3.4. Type of Medical Application for the Upper Limb

**Figure 8:**
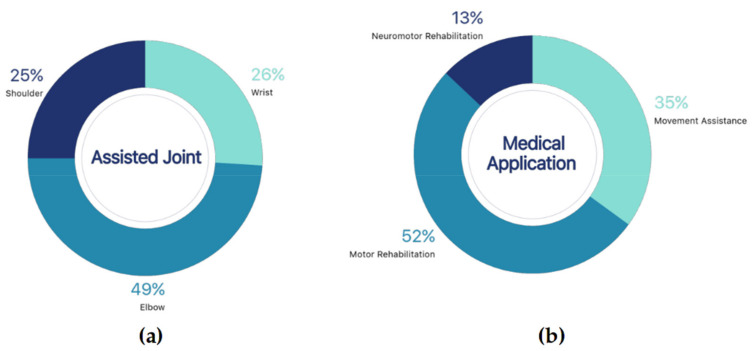
Type of medical application for the reviewed exoskeletons for the upper limbs in terms of: (**a**) joint assisted and (**b**) medical application.

Robotic exoskeletons can be used in physical therapy in different ways. The application in the healthcare field depends on the amount of assistance provided by the exoskeletons, the kind of rehabilitation or therapy implemented, and the upper limb joints available for training. A summary of the above characteristics is presented in Figure 8.

Specifically, in terms of the actively assisted joint by the robotic exoskeleton (Figure 8a), 49% of the reviewed literature refers to the elbow joint. The reason behind is that the flexion-extension movement of the arm is fundamental towards the development of daily life activities (ADLs). This is followed by the development of the wrist joint (26%) and the shoulder joint (25%), which provide additional movement of the upper limbs. However, it can be seen that: (i) due to the increasing number of people with disabilities, these machines have a major role to improve life quality for millions of people; and (ii) these systems help both people with disabilities and their caregivers by contributing to reduce the physical effort of the rehabilitation staff [93]. Below are the main applications of robotic exoskeletons targeting the upper limbs, in relation to their rehabilitation role.

#### 3.4.1. Movement Assistance

The 35% of reviewed articles were identified as contributing to the medical field as active assistance to patient mobility (Figure 8b). This means that the robotic exoskeleton is acting as functional support to perform the upper limb motion affected by pathologies, but there is no direct monitoring of the impact in terms of rehabilitation. However, the long-term benefits of movement assistance are substantial, as they improve the physical and mental health of patients who wish to recover their mobility [42,94].

Typical examples of this type of system are shown in [24,38], where the proposed designs were used to provide assistance to the patient’s movement while performing day-to-day activities. In other applications [28], exoskeletons help to reduce involuntary limb movements, which improve quality of life of patients.

The results shown in [32], has demonstrated that robotic exoskeletons for motion assistance can gradually evolve into complete rehabilitation systems. It was highlighted that the predictive features used in the control method have the potential to (i) improve the human-exoskeleton interaction when the system acts in enhancing mode, and (ii) provide greater user-friendliness for home use cases. However, more complexities emerge when considering use for rehabilitation purposes.

#### 3.4.2. Motor Rehabilitation

Robotic exoskeletons that directly target upper-limb motor rehabilitation, either at the muscular or musculoskeletal level, represent 52% of the total articles included in the review (Figure 8b). For this reason, the recovery of motor function and rehabilitation of the upper limbs represents the main medical motivation when designing and performing robotic exoskeletons [95].

The development shown in [23] underlined the improvements that can be made to the exoskeleton to meet the criteria for motor rehabilitation, emphasizing ergonomic design and improved assessment of the systems that compose the joints. The muscle tone assessments validated the development as an effective device for maintaining the physical condition of the upper limbs. Similarly, it was shown that repetitive, exoskeleton-guided physical activity can provide direct benefits in the early stages of rehabilitation by using passive or passive-active systems [25,36,48,49]. In the future, the practical effect of the rehabilitation training can be improved by changing the mechanical structure of several devices shown here.

Other articles indicate the applicability of the system to motor rehabilitation in presence of diverse motor restrictions [26,29], turning these devices as ideal candidates to be used in rehabilitation centres. It is underlined the aim of increasing the amount of motor practice carried out with assistive technologies while reducing the workload of the physiotherapists.

Several systems have reported the implementation of the motor rehabilitation function by learning the movement path through repetitive actions of the musculoskeletal system [39,43,45,47], which are able to strengthen patients’ motor abilities. Some proposed developments have focused their efforts on assisting early rehabilitation processes [40,51], benefiting or complementing future rehabilitation plans carried out with more traditional techniques.

In-depth studies have shown some devices that can assist both the rehabilitation process and the measurement of users’ physiological parameters in the rehabilitation process, making this approach more comprehensive [31]. Specifically in [37], the proposed device was designed to help stroke survivors improve their motor function without being equipped as a neurorehabilitation system. It was demonstrated that the presented exoskeleton can be applied in passive rehabilitation training and in daily life support of patients with weak upper limbs, and in [50].

#### 3.4.3. Neuromotor Rehabilitation

Studies reporting diagnostic and neuromotor rehabilitation aids are more scarce than other types of advances, being included in only 13% of the documents (Figure 8b). Developments such as [22,33,41] have highlighted that diverse control modes present in robotic exoskeletons allowed caregivers to implement different rehabilitation protocols with a single device for elderly populations or patients suffering from motor disabilities associated with neurological disorders.

Other devices help the neuromotor rehabilitation function by making use of neuro-interfaces, as proposed in [27] where the used methods can help to restore the link between the brain and the motor system after a disruption caused by pathologies such as stroke or apoplexy. These interfaces were able to identify some of the patterns of the brain waveforms, which made it possible to activate or adapt the support function of the wearable robot, enabling, for example, intense training of the function of the affected limb or accelerating the patient’s neurological recovery. In the same vein, in [46] the specific use of EEG measurement for the early pattern detection that can reveal traces of the neurological effect of the motor assistance made by the robotic exoskeleton was proposed.

Devices with dual or leader-follower architectures are essential for neuromotor rehabilitation. Specifically, in [34] it was remarked that patients can obtain training with cooperative movements between the affected and unaffected arm through tactile and virtual feedbacks.

The role of training with robotic systems oriented to neurorehabilitation is demonstrated by different models of commercial devices developed for this purpose [96,97]. It must be considered that robotic exoskeletons are not only capable of providing highly reproducible, repeatable, and accurate movements, but can also accurately and precisely provide information about the performance of the movements, making these devices usable in the rehabilitation process [98].

## 4. Discussion

Upper limb rehabilitation systems based on robotic exoskeletons have increasingly gained ground in non-engineering areas. It is important to strive for a multidisciplinary approach to make the technology more accessible, extending its potential applicability to the general public [13].

From the mechanical standpoint, the design of exoskeletons must be oriented towards more flexible, adaptable, wearable, and lightweight structures, as some of the devices do not yet fully meet these criteria [23,27,34,45,48,49,52,53]. As for the materials used for the functional structures of the devices, the use of 3D printed plastics is recommended due to rapid prototyping for user comfort, such as those evidenced in [22,26,29,33,36,42,46,48], or in a mix with low weight metal alloys [24,31,39,41,45,47,55]. The incorporation of more materials approved for medical use should be actively sought, such as [42]. Wire-actuated and soft-structured exoskeletons are also desired in rehabilitation processes because of their lightness and performance [49,50,51].

In terms of portability, many of the exoskeletons addressed in this review do not clearly or explicitly states about the power supply of the device, which makes it difficult to establish the impact of batteries or other power sources on the current design of these robotic rehabilitation devices. Beyond that, there is insufficient information about the power consumption of rehabilitation systems and their interconnection with the outside world through various communication technologies (IoT devices, wireless networks, etc.), thereby limiting the scope of this review. From this, the need for more reports on these critical aspects of portable robotic rehabilitation systems is evident, and for further scientific development in the area.

Some architectures that involve the simultaneous operation of more than one exoskeletal system or that show reflex behaviour are highlighted [27,34,49]. However, there are some drawbacks as some devices can be expensive and are not available on a large scale, making their acquisition and deployment difficult. As for the operation modes of the exoskeletons, the inclusion of several methods has been shown to improve patient recovery, and therefore a constant development of new devices with a multi-mode approach is suggested [22,23,30,35,44,46,49,55].

The collection of patients’ physiological parameters is a core element in the development of exoskeleton-based robotic systems, as these features can lead to significant improvements in robot control [22,26,29,32,43,48,54] or to improved, objective assessment demonstrating the rehabilitation process and positive effects for users [27,41,50]. It is demonstrated that the technological capabilities of robots not only guide more precise movements in rehabilitation environments but provide a fundamental and objective assessment tool.

Focusing on information collection and fusion techniques applied to the development of exoskeletal systems, it is important to note that EMG-based technologies provide reliable results, so their adoption is a cornerstone for the development of future devices. With this in mind, data fusion techniques are a key issue in the evolution of exoskeletons, as they allow the extraction of a wider set of characteristics and functional ranges from various devices [38,42,45,46,52,53,54,55].

The clinical validation of these technologies is crucial, as this paves the way to the inclusion of such technologies as support in the rehabilitation process [40]. It is concluded that clinical validation is an aspect under exploration for rehabilitation oriented mobile or wearable devices [36].

AI has provided the basis for the development of more reliable systems. Zaroug [44] emphasises that future research tends to develop exoskeletons that automatically adapt to the environment or user, actively learning from input experiences. This shows the need to create integration methods for intelligent systems and specialised hardware that cover in a global, comprehensive way the users’ needs [22,33,46,47,48,51]. Several documents of this review show natural variations of the patients or users, both within and between subjects. This inherent variability is one of the major reasons for which AI-based control systems are best suited to handle the complexities of the interaction between humans and robotic rehabilitation systems.

Considering the developments involving self-adaptive AI-based systems, independently of the used technique [27,28,46], paves the way for the future development of autonomous devices that are able to adapt to the user as they progress in the rehabilitation process. A change in the wearer’s physiological kinematics or kinetics, or their interaction with the rehabilitation device, can be interpreted as a sign of their improvement from the rehabilitation process, or a sign of a negative compensatory strategy that needs to be corrected and assessed by a specialist [99]. However, this research area should be expanded to include other intelligent control systems that allow verification and approval with well-established medical and clinical criteria.

Additionally, it is noteworthy the use of AI-based signal processing or control techniques that may bring with it some design limitations, as current AI systems may still suffer from poor explainability [100], explicit or implicit bias, or other problems related to the machine learning models [101,102], factors that may or may not manifest in a predictable way when it comes to the interaction between a human user or patient and the rehabilitation system. In view of the above, it is advisable to invest further efforts and developments in explainable AI systems that allow for a complete understanding of learning models when they are involved in critical systems such as robotic rehabilitation devices.

Finally, further exploration is recommended in approaches related to neuromotor rehabilitation and EEG signal acquisition systems, since while some advances address the topic [22,27,33,34,41,46], exoskeletons with application in motor rehabilitation are more common. It is highlighted that the addition of new virtual systems can increase neuronal plasticity in a controlled rehabilitation environment, so it is necessary to consider extending the scope to systems outside the robotic exoskeletons [103].

It can be stated that existing robotic exoskeletons have shown significant applications to upper limb rehabilitation. Beyond that, it has been demonstrated through the reviewed literature that the different robotic rehabilitation devices provide advantages for patients when directly compared to more traditional rehabilitation techniques, allowing healthcare specialists to have a tool which not only enhances patient progress, but also provides clear and objective information about the effectiveness and improvement of the rehabilitation workflow.

However, it becomes evident that there is still a need of further research to improve the mechanical structure of these devices and to implement intelligent algorithms in order to provide compact and wearable tools really usable in the neuromotor and musculoskeletal rehabilitation. There is also a need for the integration of greater expertise across multiple disciplines, and the inclusion of medical judgement from therapists or rehabilitation specialists to provide critical feedback regarding the assessment and validation of patients when using robotic rehabilitation systems. This allows the clinical evaluation of technological developments to be relevant, effective and under standardised protocols by qualified professionals [104].

## 5. Conclusions

In the last two decades, robotic exoskeletons for the upper limbs have gained great scientific interest in the areas of engineering and healthcare. They are able to provide assistance and quantitative assessments during the rehabilitation of the upper limbs. In addition, a major boost has been given by the deployment of such devices to hospitals and rehabilitation centres.

As indicated in the analysis, the researchers’ interest is focused on the development of exoskeletons based on control techniques embedding AI, covering 87% of the reviewed studies. The design of improved control strategies for robotic exoskeletons by merging information from different sources was driven forward in the last five years.

The focus is on signal processing and control systems based on ANNs (33%) and other mixed intelligent techniques (50%), as they provide better results compared to classical control strategies. The effectiveness of the processing and control systems can be improved, but requires additional research efforts to implement algorithms able to detect the real patient’s intention.

The rapid evolution of robotics and AI is radically changing the traditional rehabilitation treatments of patients. Eighty-nine percent of the studies reviewed show that the application of medical technology to physical therapy processes speeds up or improves the patient’s recovery process when compared to rehabilitation programs based solely on physical therapy sessions. Sixteen percent of studies corresponding to mobile or portable exoskeletons are able to provide direct assistance to the treatment of neuromotor injuries. This will potentially contribute to the partial or total treatment of high severity pathologies in an effective way.

The implementation of rehabilitation programs based on portable technological devices offers a way to redefine physical therapies, which requires immediate actions for the rapid adoption of such devices in the clinical practice.

In contrast, there are also some key elements that need to be investigated in further detail. As a roadmap, the priority should be given to developing robotic rehabilitation exoskeletons that are both ergonomic and portable, as some of them still rely on additional physical support structures. The existence of an increasing number of fully wearable devices is highlighted (53%), where the use of this type of exoskeleton provides increased mobility and allows patients to improve their neuromotor skills during ADLs. In this context, functional rehabilitation of the upper extremities can offer a wide range of solutions even outside the clinical settings.

The reviewed studies point out the need to continue ongoing research in order to provide a more integrated and comprehensive framework based on the above-mentioned technologies. This will also increase their reliability and their adoption in the rehabilitation units. Lastly, there is also a critical need to develop fully explainable or deducible Artificial Intelligence systems, which will enable synergistic and transparent work between new and improved rehabilitation systems and healthcare professionals. As the AI field is constantly evolving, it is expected that recent advances in the explainability of machine learning and deep learning models will be incorporated by the researchers in the area of engineering and rehabilitation.

The current status of upper limb rehabilitation systems based on portable robotic exoskeletons shows that some relevant gaps should be filled in, where intelligent control and information processing systems can play key roles. In addition, with the improvement of materials and the incorporation of better mechanical designs, the capabilities of exoskeletons can be largely improved.

## Figures and Tables

**Table 1:** Explicit formulation of the search queries for each database.

Database	Search Query
Scopus	TITLE-ABS-KEY ((“powered exoskeleton” OR “robotic exoskeleton” OR “active exoskeleton” OR exosuit OR “powered ortho*” OR “robotic ortho*” OR “active ortho*”) AND ((up* OR “upper limb” OR arm OR forearm OR shoulder OR elbow) AND NOT (low* OR hip OR gait OR vision OR theoretical OR walk*)) AND (rehabilitation OR “physical therapy” OR impair* OR health)) AND (intellig* OR adaptiv* OR netw* OR “artificial neur*” OR ANN OR learn*) AND (wearable OR mobile OR portable) AND (LIMIT-TO (SUBJAREA, “ENGI”) OR LIMIT-TO (SUBJAREA, “COMP”) OR LIMIT-TO (SUBJAREA, “MEDI”)) AND (LIMIT-TO (PUBYEAR, 2020) OR LIMIT-TO (PUBYEAR, 2019) OR LIMIT-TO (PUBYEAR, 2018) OR LIMIT-TO (PUBYEAR, 2017) OR LIMIT-TO (PUBYEAR, 2016))
IEEE Xplore	((exoskeleton OR exosuit OR ortho*) AND (upper OR arm OR forearm OR shoulder OR elbow) AND (rehabilitation OR “physical therapy” OR impairment OR health) AND (intellig* OR adaptive OR netw* OR “artificial neur*” OR ANN OR learn*) AND (wearable OR mobile OR portable) NOT (low* OR hip OR gait OR walk*))
Web of Science	TS=((exoskeleton OR exosuit OR ortho*) AND (upper OR arm OR forearm OR shoulder OR elbow) AND (rehabilitation OR “physical therapy” OR impair* OR health) AND (intellig* OR adaptiv* OR netw* OR “artificial neur*” OR ANN OR learn*) AND (wearable OR mobile OR portable) NOT (low* OR hip OR gait OR walk* OR vision OR theoretical)) AND PY=(2020 OR 2019 OR 2018 OR 2017 OR 2016)
PubMed	((exoskeleton OR exosuit OR ortho*) AND (upper OR arm OR forearm OR shoulder OR elbow) AND (rehabilitation OR “physical therapy” OR impair* OR health) AND (intellig* OR adaptiv* OR netw* OR “artificial neur*” OR ANN OR learn*) AND (wearable OR mobile OR portable) NOT (low* OR hip OR gait OR walk*)) AND ((“2016”[Date—Publication]: “2020”[Date—Publication]))

**Table 2:** General summary of the main characteristics of the studies included in the review.

No.	Ref.	Year	MF	Exoskeleton Type	Portability	Used AI Techniques	Operation Modes	Medical App.	DoF-Joint
1	[22]	2016	Dev.	Hard: 3DPL/ME	Wearable	Artificial Neural Networks	AC/PA/RE	NMR	(1)-Wrist
2	[23]	2016	Dev.	Hard: PL/ME	Semi-mobile	Adaptive Finite State Machines	AC/RE /OT	MR	(2)-Shoulder (1)-Elbow
3	[24]	2017	Dev.	Hard: ME	Wearable	Adaptive Torque Compensation	AC/PA	AA/MR	(3)-Shoulder (2)-Elbow
4	[25]	2017	Dev.	Not Shown	Mobile	PD + Intelligent Active Force (AFC) + Particle Swarm Optimization	AC	MR	(1)-Shoulder (1)-Elbow
5	[26]	2017	Dev.	Hard: 3DPL/ME	Wearable	Fuzzy Logic via Artificial Neural Networks	AC	MR	(1)-Elbow
6	[27]	2017	Dev.	Dual Arm Hard: ME	Wearable	Adaptive Algorithms + Artificial Neural Networks	AC	NMR	(4)-Back (3)-Shoulder (1)-Elbow (2)-Wrist
7	[28]	2017	Com.	Semi-hard: PL	Wearable	Adaptive Fuzzy PID Controller	AC/OT	AA	(1)-Wrist/Elbow
8	[29]	2017	Dev.	Hard: 3DPL	Wearable	Adaptive Admittance and Proportional Derivative Cont.	AC	MR	(1)-Wrist
9	[30]	2018	Mix.	Mixed	Mixed	Mixed	AC/RE /OT	Mixed	Mixed
10	[31]	2018	Dev.	Hard: ME	Mobile	Adaptive Sliding Modes + Adaptive Proportional Derivative	AC	MR	(3)-Shoulder (2)-Elbow
11	[32]	2018	Dev.	Hard: PL	Wearable	Active Learning Mapping via KNN classifier	AC	AA	(1)-Wrist
12	[33]	2018	Dev.	Hard: 3DPL	Mobile	Reinforcement Learning via Artificial Neural Networks and Proximal Policy Optimization	PA	NMR	(2)-Shoulder (1)-Elbow
13	[34]	2018	Dev.	Dual Arm Hard: PL/ME	Semi-mobile	Impedance control via cascaded loop	AC	AA/NMR	(1)-Elbow (1)-Wrist
14	[35]	2018	Dev.	Mixed	Mixed	Adaptive controllers via mixed techniques.	AC/RE /OT	Mixed	Mixed
15	[36]	2019	Dev.	Hard: 3DPL/ME	Wearable	Sliding Mode anti-interference controller	PA	AA/MR	(1)-Elbow (1)-Wrist
16	[37]	2019	Dev.	Hard: PL/ME	Wearable	Adaptive Algorithms + Artificial Neural Networks	AC/PA	AA/MR	(1)-Elbow (5)-Passive
17	[38]	2019	Dev.	Not Shown	Mobile	Recurrence Quantification Analysis (RQA)	PA	AA	Not Shown
18	[39]	2019	Dev.	Hard: ME	Wearable	Hierarchical Support Vector Machines	AC	MR	(2)-Elbow (1)-Wrist
19	[40]	2019	Dev.	Hard: PL/ME	Wearable	Radial Basis Function Artificial Neural Network	AC	MR	(1)-Shoulder (1)-Elbow (1)-Wrist
20	[41]	2019	Dev.	Hard: ME	Wearable	Adaptive Feedforward control scheme	AC/PA	AA/MR	(1)-Elbow
21	[42]	2019	Dev.	Hard: 3DPL	Wearable	Fuzzy Logic via Artificial Neural Networks	AC	AA	(2)-Elbow (1)-Wrist
22	[43]	2019	Dev.	Hard: ME	Wearable	Backpropagated Neural Network	AC	MR	(2)-Elbow (1)-Wrist
23	[44]	2019	Mix.	Mixed	Mixed	Mixed	AC/RE /OT	Mixed	Mixed
24	[45]	2019	Dev.	Hard: ME	Semi-mobile	Heuristically-tuned Proportional Derivative cont.	AC	MR	(1)-Elbow
25	[46]	2019	Dev.	Hard: 3DPL/ME	Wearable	Adaptive Algorithms + Artificial Neural Networks	AC/RE /OT	AA/MR/NMR	(1)-Elbow
26	[47]	2020	Dev.	Hard: ME	Wearable	Artificial Neural Networks	AC	MR	(2)-Elbow (1)-Wrist
27	[48]	2020	Dev.	Hard: 3DPL	Semi-mobile	Backpropagated Neural Network	AC	AA/MR	(1)-Elbow
28	[49]	2020	Dev.	Dual Arm Hard: ME	Semi-mobile	Fuzzy Sliding Mode controller	AC/OT	AA/MR	(3)-Shoulder (1)-Elbow
29	[50]	2020	Dev.	Soft	Wearable	Artificial Neural Networks + Sliding Mode controller	AC	MR	(1)-Shoulder (1)-Elbow
30	[51]	2020	Dev.	Soft	Wearable	Multivariate Multiple Regression via Artificial Neural Networks	AC	MR	(1)-Shoulder (1)-Elbow
31 32	[52] * [53] *	2017 2019	Dev.	Full-Body Hard: ME	Semi-mobile	Impedance control via cascaded loop + mixed	AC/RE /OT	AA/MR	(10)-Upper Body (30)-Full Body
33	[54] *	2019	Dev.	Full-Body Hard: 3DPL/ME	Semi-mobile	Mixed	AC/PA/RE	MR	(12)-Full Body
34	[55] *	2017	Dev.	Dual Arm Hard: 3DPL/ME	Mobile	Impedance control via cascaded loop + mixed	AC/RE /OT	MR	(14)-Upper Body

* Complementary studies that were included due to their relevance. However, these documents are not part of the statistical analysis, since they were not retrieved using the search query and the PRISMA methodology used for the development of this review.

## Data Availability

Not applicable.

## References

[1] World Health Organization, The World Bank (2011). World Report on Disability.

[2] Mathers C., Fat D.M., Boerma J.T., World Health Organization (2008). The Global Burden of Disease: 2004 Update.

[3] Mcfarlane L., Mclean J. (2003). Education and training for direct care workers. Soc. Work Educ..

[4] Young J., Forster A. (2007). Review of stroke rehabilitation. BMJ.

[5] Adamson J., Beswick A., Ebrahim S. (2004). Is stroke the most common cause of disability?. J. Stroke Cerebrovasc. Dis..

[6] Katan M., Luft A. (2018). Global Burden of Stroke. Semin. Neurol..

[7] Innocenti T., Ristori D., Miele S., Testa M. (2018). The management of shoulder impingement and related disorders: A systematic review on diagnostic accuracy of physical tests and manual therapy efficacy. J. Bodyw. Mov. Ther..

[8] Azma K., RezaSoltani Z., Rezaeimoghaddam F., Dadarkhah A., Mohsenolhosseini S. (2018). Efficacy of tele-rehabilitation compared with office-based physical therapy in patients with knee osteoarthritis: A randomized clinical trial. J. Telemed. Telecare.

[9] Longley V., Peters S., Swarbrick C., Bowen A. (2019). What factors affect clinical decision-making about access to stroke rehabilitation? A systematic review. Clin. Rehabil..

[10] Vukobratovic M.K. (2007). When were acitve exoskeletons actually born?. Int. J. Hum. Robot..

[11] Dellon B., Matsuoka Y. (2007). Prosthetics, exoskeletons, and rehabilitation [Grand Challenges of Robotics]. IEEE Robot. Autom. Mag..

[12] Pons J.L. (2010). Rehabilitation Exoskeletal Robotics. IEEE Eng. Med. Biol. Mag..

[13] Gorgey A.S. (2018). Robotic exoskeletons: The current pros and cons. World J. Orthop..

[14] Wang Q., Markopoulos P., Yu B., Chen W., Timmermans A. (2017). Interactive wearable systems for upper body rehabilitation: A systematic review. J. Neuroeng. Rehabil..

[15] Bouteraa Y., Ben Abdallah I. Exoskeleton robots for upper-limb rehabilitation. Proceedings of the 13th International Multi-Conference on Systems, Signals and Devices, SSD 2016.

[16] Balasubramanian S., Wei R., Perez M., Shepard B., Koeneman E., Koeneman J., He J. (2008). RUPERT: An exoskeleton robot for assisting rehabilitation of arm functions. Virtual Rehabil..

[17] Mekki M., Delgado A.D., Fry A., Putrino D., Huang V. (2018). Robotic Rehabilitation and Spinal Cord Injury: A Narrative Review. Neurotherapeutics.

[18] Gilhooly R. Exoskeletons Await in Work/Care Closet. http://www.japantimes.co.jp/life/2012/06/17/general/exoskeletons-await-in-workcare-closet.

[19] Manna S.K., Dubey V.N. (2018). Comparative study of actuation systems for portable upper limb exoskeletons. Med. Eng. Phys..

[20] Smith J.E. (2012). Self-adaptative and coevolving memetic algorithms. Stud. Comput. Intell..

[21] Liberati A., Altman D.G., Tetzlaff J., Mulrow C., Gøtzsche P.C., Ioannidis J.P.A., Clarke M., Devereaux P.J., Kleijnen J., Moher D. (2009). The PRISMA statement for reporting systematic reviews and meta-analyses of studies that evaluate health care interventions: Explanation and elaboration. PLoS Med..

[22] Sangha S., Elnady A.M., Menon C. A compact robotic orthosis for wrist assistance. Proceedings of the 2016 6th IEEE International Conference on Biomedical Robotics and Biomechatronics (BioRob).

[23] Chonnaparamutt W., Supsi W. (2016). SEFRE: Semiexoskeleton Rehabilitation System. Appl. Bionics Biomech..

[24] Sui D., Fan J., Jin H., Cai X., Zhao J., Zhu Y. Design of a wearable upper-limb exoskeleton for activities assistance of daily living. Proceedings of the 2017 IEEE International Conference on Advanced Intelligent Mechatronics (AIM).

[25] Abdul Majeed A.P.P., Taha Z., Mohd Khairuddin I., Wong M.Y., Abdullah M.A., Mohd Razman M.A. (2017). The control of an upper-limb exoskeleton by means of a particle swarm optimized active force control for motor recovery. IFMBE Proc..

[26] Tageldeen M.K., Perumal N., Elamvazuthi I., Ganesan T. Design and control of an upper arm exoskeleton using Fuzzy logic techniques. Proceedings of the 2016 2nd IEEE International Symposium on Robotics and Manufacturing Automation (ROMA).

[27] Seeland A., Tabie M., Kim S.K., Kirchner F., Kirchner E.A. Adaptive multimodal biosignal control for exoskeleton supported stroke rehabilitation. Proceedings of the 2017 IEEE International Conference on Systems, Man, and Cybernetics (SMC).

[28] Shamroukh M., Chacko A., Kalaichelvi V., Kalimullah I.Q., Barlingay S.S., Chattopadhyay A.B. Evaluation of control strategies in semi-active orthosis for suppression of upper limb pathological tremors. Proceedings of the 2017 International Conference on Innovations in Electrical, Electronics, Instrumentation and Media Technology (ICEEIMT).

[29] Lambelet C., Lyu M., Woolley D., Gassert R., Wenderoth N. The eWrist—A wearable wrist exoskeleton with sEMG-based force control for stroke rehabilitation. Proceedings of the 2017 International Conference on Rehabilitation Robotics, ICORR 2017.

[30] Rehmat N., Zuo J., Meng W., Liu Q., Xie S.Q., Liang H. (2018). Upper limb rehabilitation using robotic exoskeleton systems: A systematic review. Int. J. Intell. Robot. Appl..

[31] Rosales Luengas Y., López-Gutiérrez R., Salazar S., Lozano R. (2018). Robust controls for upper limb exoskeleton, real-time results. Proc. Inst. Mech. Eng. Part I J. Syst. Control Eng..

[32] Siu H.C., Arenas A.M., Sun T., Stirling L.A. (2018). Implementation of a surface electromyography-based upper extremity exoskeleton controller using learning from demonstration. Sensors.

[33] Di Febbo D., Ambrosini E., Pirotta M., Rojas E., Restelli M., Pedrocchi A.L.G., Ferrante S. Does Reinforcement Learning outperform PID in the control of FES-induced elbow flex-extension?. Proceedings of the 2018 IEEE International Symposium on Medical Measurements and Applications (MeMeA).

[34] Zhang S., Fu Q., Guo S., Fu Y. (2018). Coordinative Motion-based Bilateral Rehabilitation Training System with Exoskeleton and Haptic Devices for Biomedical Application. Micromachines.

[35] Miao Q., Zhang M., Cao J., Xie S.Q. (2018). Reviewing high-level control techniques on robot-assisted upper-limb rehabilitation. Adv. Robot..

[36] Zhao Z., Li X., Hao L. Research on the Control Method of a Rehabilitation Exoskeleton Robot for Passive Training on Upper-Limbs of Stroke Patients. Proceedings of the 2018 IEEE 8th Annual International Conference on CYBER Technology in Automation, Control, and Intelligent Systems (CYBER).

[37] Liu Y., Guo S. Design of a Novel Wearable Power-assist Exoskeleton Device. Proceedings of the 2018 13th World Congress on Intelligent Control and Automation (WCICA).

[38] Chen L., Zhang C., Liu Z., Zhang T. Evaluation of Muscle Fatigue Based on CRP and RQA for Upper Limb Exoskeleton. Proceedings of the 2018 Chinese Automation Congress (CAC).

[39] Wang W., Qin L., Yuan X., Ming X., Sun T., Liu Y. (2019). Bionic control of exoskeleton robot based on motion intention for rehabilitation training. Adv. Robot..

[40] Guo S., Gao W., Bu D. Radial Basis Function Neural Network-based Control Method for a Upper Limb Rehabilitation Robot. Proceedings of the 2019 IEEE International Conference on Mechatronics and Automation (ICMA).

[41] Asokan A., Vigneshwar M. Design and Control of an EMG-based Low-cost Exoskeleton for Stroke Rehabilitation. Proceedings of the 2019 Fifth Indian Control Conference (ICC) 2019.

[42] Al Bakri A., Lezzar M.Y., Alzinati M., Mortazavi K., Shehieb W., Sharif T. Intelligent Exoskeleton for Patients with Paralysis. Proceedings of the 2018 IEEE 9th Annual Information Technology, Electronics and Mobile Communication Conference (IEMCON).

[43] Lei Z. (2019). An upper limb movement estimation from electromyography by using BP neural network. Biomed. Signal Process. Control.

[44] Zaroug A., Proud J.K., Lai D.T.H., Mudie K., Billing D., Begg R. (2019). Overview of Computational Intelligence (CI) Techniques for Powered Exoskeletons. Computational Intelligence in Sensor Networks.

[45] Taha Z., Abdul Majeed A.P.P., Abdullah M.A., Azmi K.Z.M., Bin Zakaria M.A., Ghani A.S.A., Hassan M.H.A., Razman M.A.M. (2019). The control of an upper extremity exoskeleton for stroke rehabilitation by means of a hybrid active force control. Proceedings of the Advances in Intelligent Systems and Computing.

[46] Vélez-Guerrero M.A., Callejas-Cuervo M. Data Acquisition and Control Architecture for Intelligent Robotic Exoskeletons in Rehabilitation. Proceedings of the 7th IEEE International Conference on E-Health and Bioengineering—EHB 2019.

[47] Wang W., Li H., Xiao M., Chu Y., Yuan X., Ming X., Zhang B. (2020). Design and verification of a human–robot interaction system for upper limb exoskeleton rehabilitation. Med. Eng. Phys..

[48] Li X., Liu S., Chang Y., Li S., Fan Y., Yu H. (2020). A Human Joint Torque Estimation Method for Elbow Exoskeleton Control. Int. J. Humanoid Robot..

[49] Chen C.T., Lien W.Y., Chen C.T., Twu M.J., Wu Y.C. (2020). Dynamic Modeling and Motion Control of a Cable-Driven Robotic Exoskeleton with Pneumatic Artificial Muscle Actuators. IEEE Access.

[50] Samper-Escudero J.L., Gimenez-Fernandez A., Sanchez-Uran M.A., Ferre M. (2020). A Cable-Driven Exosuit for Upper Limb Flexion Based on Fibres Compliance. IEEE Access.

[51] Varghese R.J., Lo B.P.L., Yang G.Z. (2020). Design and Prototyping of a Bio-Inspired Kinematic Sensing Suit for the Shoulder Joint: Precursor to a Multi-DoF Shoulder Exosuit. IEEE Robot. Autom. Lett..

[52] Kumar S., Simnofske M., Bongardt B., Müller A., Kirchner F. Integrating mimic joints into dynamics algorithms: Exemplified by the hybrid recupera exoskeleton. Proceedings of the Advances in Robotics.

[53] Kumar S., Wöhrle H., Trampler M., Simnofske M., Peters H., Mallwitz M., Kirchner E., Kirchner F. (2019). Modular Design and Decentralized Control of the Recupera Exoskeleton for Stroke Rehabilitation. Appl. Sci..

[54] Christensen S., Bai S., Rafique S., Isaksson M., O’Sullivan L., Power V., Virk G.S. (2019). AXO-SUIT—A Modular Full-Body Exoskeleton for Physical Assistance.

[55] Kim B., Deshpande A.D. (2017). An upper-body rehabilitation exoskeleton Harmony with an anatomical shoulder mechanism: Design, modeling, control, and performance evaluation. Int. J. Rob. Res..

[56] Van Eck N.J., Waltman L. (2010). Software survey: VOSviewer, a computer program for bibliometric mapping. Scientometrics.

[57] Reanaree P., Pintavirooj C. Exoskeleton Suit Supports the Movement. Proceedings of the 2018 11th Biomedical Engineering International Conference (BMEiCON).

[58] Dollar A.M., Herr H. (2008). Lower Extremity Exoskeletons and Active Orthoses: Challenges and State-of-the-Art. IEEE Trans. Robot..

[59] Abdul Majeed A.P.P., Taha Z., Abdullah M.A., Azmi K.Z.M., Bin Zakaria M.A. (2018). The control of an upper extremity exoskeleton for stroke rehabilitation: An active force control scheme approach. Adv. Robot. Res..

[60] Nag P.K., Mission R., Sinha A.G.K., Goswami A. (2014). Introduction and Classification of Therapeutic Exercise.

[61] Gull M.A., Bai S., Bak T. (2020). A review on design of upper limb exoskeletons. Robotics.

[62] De Vries A., De Looze M. (2019). The Effect of Arm Support Exoskeletons in Realistic Work Activities: A Review Study. J. Ergon..

[63] Lee S.H., Park G., Cho D.Y., Kim H.Y., Lee J.Y., Kim S., Park S.B., Shin J.H. (2020). Comparisons between end-effector and exoskeleton rehabilitation robots regarding upper extremity function among chronic stroke patients with moderate-to-severe upper limb impairment. Sci. Rep..

[64] Pang Z., Wang T., Wang Z., Yu J., Sun Z., Liu S. (2020). Design and analysis of a wearable upper limb rehabilitation robot with characteristics of tension mechanism. Appl. Sci..

[65] Mallwitz M., Will N., Teiwes J., Kirchner E.A. The CAPIO Active Upper Body Exoskeleton and Its Application for Teleoperation. Proceedings of the 13th Symposium on Advanced Space Technologies in Robotics and Automation. ESA/Estec Symposium on Advanced Space Technologies in Robotics and Automation (ASTRA-2015).

[66] McPhee J. (2019). Integration of Machine Learning with Dynamics and Control: From Autonomous Cars to Biomechatronics. CSME Bull..

[67] Novak D., Riener R. (2015). Control strategies and artificial intelligence in rehabilitation robotics. AI Mag..

[68] Losey D.P., McDonald C.G., Battaglia E., O’Malley M.K. (2018). A Review of Intent Detection, Arbitration, and Communication Aspects of Shared Control for Physical Human–Robot Interaction. Appl. Mech. Rev..

[69] Bonato P. (2005). Advances in wearable technology and applications in physical medicine and rehabilitation. J. Neuroeng. Rehabil..

[70] Nayak S., Kumar Das R. (2016). Application of Artificial Intelligence (AI) in Prosthetic and Orthotic Rehabilitation. Service Robotics.

[71] Samper-Escudero J.L., Contreras-González A.F., Ferre M., Sánchez-Urán M.A., Pont-Esteban D. (2020). Efficient Multiaxial Shoulder-Motion Tracking Based on Flexible Resistive Sensors Applied to Exosuits. Soft Robot..

[72] Belda K., Böhm J. (2006). Adaptive Predictive Control for Simple Mechatronic Systems. Proceedings of the 10th WSEAS International Conference on Systems.

[73] Szuster M., Hendzel Z. (2018). Intelligent Optimal Adaptive Control for Mechatronic Systems.

[74] Marchal-Crespo L., Reinkensmeyer D.J. (2009). Review of control strategies for robotic movement training after neurologic injury. J. Neuroeng. Rehabil..

[75] Mounis S.Y.A., Azlan N.Z., Sado F. (2019). Assist-as-needed control strategy for upper-limb rehabilitation based on subject’s functional ability. Meas. Control (UK).

[76] Benveniste A., Wilson S.S., Metivier M., Priouret P. (2012). Adaptive Algorithms and Stochastic Approximations.

[77] Gambhire S.J., Kishore D.R., Londhe P.S., Pawar S.N. (2020). Review of sliding mode based control techniques for control system applications. Int. J. Dyn. Control.

[78] Babaiasl M., Goldar S.N., Barhaghtalab M.H., Meigoli V. Sliding mode control of an exoskeleton robot for use in upper-limb rehabilitation. Proceedings of the 2015 3rd RSI International Conference on Robotics and Mechatronics (ICROM).

[79] Anam K., Al-Jumaily A.A. (2012). Active exoskeleton control systems: State of the art. Procedia Eng..

[80] Esmaeili B., Beyramzad J., Seyyedrasuli M., Noorani M.R.S., Ghanbari A. Using fuzzy neural network sliding mode control for human-exoskeleton interaction forces minimization. Proceedings of the 2018 IEEE International Conference on Mechatronics and Automation (ICMA).

[81] Rahmani M., Rahman M.H. (2019). An upper-limb exoskeleton robot control using a novel fast fuzzy sliding mode control. J. Intell. Fuzzy Syst..

[82] Widrow B., Lehr M.A. (1993). Adaptive neural networks and their applications. Int. J. Intell. Syst..

[83] Magoulas G.D., Vrahatis M.N. (2006). Adaptive algorithms for neural network supervised learning: A deterministic optimization approach. Int. J. Bifurc. Chaos.

[84] Kar S., Das S., Ghosh P.K. (2014). Applications of neuro fuzzy systems: A brief review and future outline. Appl. Soft Comput. J..

[85] Xu L., Vu Y., Wu Q. (1999). General Fuzzy Neural Network: Basic structure, algorithms and its applications. IFAC Proc. Vol..

[86] Kiguchi K., Tanaka T., Fukuda T. (2004). Neuro-fuzzy control of a robotic exoskeleton with EMG signals. IEEE Trans. Fuzzy Syst..

[87] Stingu P.E., Lewis F.L., Meyers R.A. (2009). Neuro-fuzzy Control of Autonomous Robotics. Encyclopedia of Complexity and Systems Science.

[88] Jiang Y., Yang C., Ma H. (2016). A Review of Fuzzy Logic and Neural Network Based Intelligent Control Design for Discrete-Time Systems. Discret. Dyn. Nat. Soc..

[89] Yang S., Han J., Xia L., Chen Y.H. (2020). An optimal fuzzy-theoretic setting of adaptive robust control design for a lower limb exoskeleton robot system. Mech. Syst. Signal Process..

[90] Ou Y., Li Z., Li G., Su C.Y. Adaptive fuzzy tracking control of a human lower limb with an exoskeleton. Proceedings of the 2012 IEEE International Conference on Robotics and Biomimetics (ROBIO).

[91] Rahmani M., Rahman M.H., Ghommam J. (2019). A 7-DoF Upper Limb Exoskeleton Robot Control Using a New Robust Hybrid Controller. Int. J. Control. Autom. Syst..

[92] Bembli S., Haddad N.K., Belghith S. (2019). Robustness Analysis of an Upper Limb Exoskeleton Controlled by Sliding Mode Algorithm. Mech. Mach. Sci..

[93] Anirudh Sharma C., Sai A.S.K., Kumar V., Prasad A., Begum R., Sharvani G.S., Manjunath A.E. Multifaceted Bio-medical applications of Exoskeleton: A review. Proceedings of the 2018 2nd International Conference on Inventive Systems and Control (ICISC).

[94] Kiguchi K. (2007). Active exoskeletons for upper-limb motion assist. Int. J. Hum. Robot..

[95] Ruiz A.F., Forner-Cordero A., Rocon E., Pons J.L. Exoskeletons for rehabilitation and motor control. Proceedings of the First IEEE/RAS-EMBS International Conference on Biomedical Robotics and Biomechatronics.

[96] Proietti T., Crocher V., Roby-Brami A., Jarrasse N. (2016). Upper-limb robotic exoskeletons for neurorehabilitation: A review on control strategies. IEEE Rev. Biomed. Eng..

[97] Onose G., Popescu N., Munteanu C., Ciobanu V., Sporea C., Mirea M.D., Daia C., Andone I., Spînu A., Mirea A. (2018). Mobile mechatronic/robotic orthotic devices to assist-rehabilitate neuromotor impairments in the upper limb: A systematic and synthetic review. Front. Neurosci..

[98] Iandolo R., Marini F., Semprini M., Laffranchi M., Mugnosso M., Cherif A., De Michieli L., Chiappalone M., Zenzeri J. (2019). Perspectives and challenges in robotic neurorehabilitation. Appl. Sci..

[99] Morgenstern J.D., Rosella L.C., Daley M.J., Goel V., Schünemann H.J., Piggott T. (2021). “AI’s gonna have an impact on everything in society, so it has to have an impact on public health”: A fundamental qualitative descriptive study of the implications of artificial intelligence for public health. BMC Public Health.

[100] Chen T., Keravnou-Papailiou E., Antoniou G. (2021). Medical analytics for healthcare intelligence—Recent advances and future directions. Artif. Intell. Med..

[101] Sherratt F., Plummer A. (2021). Understanding LSTM Network Behaviour of IMU-Based. Sensors.

[102] Monardo G., Pavese C., Giorgi I., Godi M., Colombo R. (2021). Evaluation of Patient Motivation and Satisfaction during Technology-Assisted Rehabilitation: An Experiential Review. Games Health J..

[103] Alarcón-Aldana A.C., Callejas-Cuervo M., Bo A.P.L. (2020). Upper limb physical rehabilitation using serious videogames and motion capture systems: A systematic review. Sensors.

[104] De la Tejera J.A., Bustamante-Bello R., Ramirez-Mendoza R.A., Izquierdo-Reyes J. (2021). Systematic review of exoskeletons towards a general categorization model proposal. Appl. Sci..

